# Development of a Brief Intervention for Emergency Department Attendees Presenting With Self-Harm and Co-Occurring Substance Use Problems: A Delphi Method Study

**DOI:** 10.1027/0227-5910/a000933

**Published:** 2023-11-17

**Authors:** Prianka Padmanathan, Rachel Cohen, David Gunnell, Lucy Biddle, Emma Griffith, Katie Breheny, Matt Hickman, Nik Munien, Anish Patel, Elaine Crocker, Paul Moran

**Affiliations:** ^1^ Population Health Sciences, Bristol Medical School, University of Bristol, England, UK; ^2^ Liaison Psychiatry, Avon and Wiltshire Mental Health Partnership NHS Trust, Bath, England, UK; ^3^ Welsh Government Health and Social Services Group, Social Services and Integration Directorate, Futures and Integration Division, Cardiff, Wales, UK; ^4^ National Institute for Health and Care Research Biomedical Research Centre, University Hospitals Bristol and Weston NHS Foundation Trust and the University of Bristol, England, UK; ^5^ National Institute for Health and Care Research Applied Research Collaboration West, University Hospitals Bristol and Weston NHS Foundation Trust, England, UK; ^6^ Department of Psychology, University of Bath, England, UK; ^7^ Specialised Services, Avon and Wiltshire Mental Health Partnership NHS Trust, Bath, England, UK; ^8^ National Institute for Health and Care Research Health Protection Research Unit in Behavioural Science and Evaluation, University of Bristol, England, UK; ^9^ Liaison Psychiatry, University Hospitals Bristol and Weston NHS Foundation Trust, Bristol, England, UK; ^10^ Liaison Psychiatry, North Bristol NHS Trust, Bristol, England, UK

**Keywords:** suicide, self-harm, substance use, psychosocial, intervention

## Abstract

**Abstract:** *Background:* People who present to the emergency department with self-harm and co-occurring substance use problems often have difficulty accessing effective care. *Aims:* To develop a brief psychosocial intervention for this population, which would be suitable for testing in a future randomized controlled trial. *Methods:* A modified Delphi method was used. A 34-item, 3-round, online Delphi survey was informed by a literature review and stakeholder telephone discussions (*n =* 17). Two panels consisting of people with lived experience (PWLE: *n =* 15) and people with occupational experience (PWOE: *n =* 21) participated in the survey. The threshold for consensus was a pooled agreement rate across the two panels of 80% or more. *Results:* Expert consensus was achieved for 22 items. The new intervention consists of weekly follow-up phone calls for up to 1 month, delivered by Liaison Psychiatry practitioners, in which both self-harm and substance use problems are explored and addressed, and patients are supported in accessing community services. *Limitations:* Some stakeholder ideas regarding intervention components could not be included as survey options due to anticipated difficulties with implementation. *Conclusions:* The key elements of a brief psychosocial intervention for self-harm and co-occurring substance use problems have been agreed. Feasibility testing is currently underway.

The presentation of patients to hospital with self-harm represents a key opportunity for suicide prevention. Almost one in five people presenting to hospital with self-harm go on to repeat self-harm within the following year, 1 in 25 die by suicide within the next 5 years (Carroll et al., 2014), and the highest incidence of suicide occurs in the first month after presentation (Geulayov et al., 2019). Hospital presentation can also be considered a *teachable moment*, i.e., a life event or transition during which individuals have increased motivation for behavioral change and low-intensity interventions may be more effective (McBride et al., 2003).

There is growing evidence to support the use of active contact and follow-up (including brief interventions) to prevent suicide among people presenting to hospital with an identified suicide risk (Doupnik et al., 2020; Miller et al., 2017; O’Connor et al., 2022; Stanley et al., 2018). The effectiveness of these types of interventions for people with substance use problems is, however, currently unknown. Despite their increased risk of suicide, people with substance use problems are often excluded from both self-harm research intervention studies (Hawton et al., 2016) and mental health services (Chandler & Taylor, 2021; Public Health England, 2017). Previous large randomized controlled trials, which have been specifically aimed at people with both substance use problems and suicide risk, have been compromised by limited engagement and have been unable to demonstrate a reduction in suicide-related thoughts and behaviors (Crawford et al., 2010; Morley et al., 2014). Adaptation of self-harm interventions to meet the specific needs and preferences of people with substance use problems has the potential to improve engagement and treatment outcomes. For example, it may be beneficial to incorporate tools such as motivational interviewing and contingency management, which have been extensively researched within the field of substance use treatments but are not currently used in suicide prevention interventions (Pickard & Ahmed, 2018).

In this study, we sought to combine current research evidence with the expertise of people with occupational experience (PWOE) or people with lived experience (PWLE) to inform (1) the core components of a new brief psychosocial intervention for people presenting to the emergency department with self-harm and substance use problems and (2) the design of a future randomized controlled trial of the new intervention. When using the term substance use problems, we refer to hazardous, harmful, and dependent levels of substance use (World Health Organization, 2019).

## Methods

The Delphi method is a consensus method, which includes an iterative multiround survey during which experts individually rate and provide comments on survey items based on their level of agreement with them (Hasson et al., 2000; Jones & Hunter, 1995). After each round, a summary of ratings and comments is fed back to respondents, who then complete the next round by rerating the items.

We used the modified Delphi subtype, in which preselected survey items can be informed by a variety of sources, including a systematic review or focus groups (Hasson & Keeney, 2011). Our survey was informed by a systematic review of interventions to prevent suicide and reduce self-harm among people with substance use problems (Padmanathan et al., 2020), the wider literature on self-harm risk and interventions (Hawton et al., 2016; Inagaki et al., 2019; McCabe et al., 2018; Miller et al., 2017; O’Connor et al., 2019; Stanley et al., 2018), telephone interviews with individuals with occupational and lived experience, and the authors’ clinical judgment. Where the authors’ clinical judgment was used, this primarily related to ensuring that the intervention would be feasible to implement and evaluate within National Health Service (NHS) clinical services. The rationale for decisions made prior to the survey, including the selection of items included in the survey, is provided in Electronic Supplementary Material 1 (ESM 1).

### Participant (Panel) Recruitment

Two panels were recruited. The first panel included people with occupational experience (PWOE) who had clinical, research, or service provision experience relating to suicide and/or substance use problems. The second panel included people with any lived experience (PWLE) of both substance use problems and suicidal thoughts, suicide attempts, or self-harm.

Participants were recruited to the telephone interviews using the same recruitment method as the survey (described below). Participants who completed a telephone interview were also invited to complete the survey. Of the 17 participants who completed the telephone interviews (PWOE: *n =* 10; PWLE: *n =* 7), 14 (PWOE: *n =* 8; PWLE: *n =* 6) also completed the survey. Once sufficient information had been obtained from the telephone interviews to inform the survey, additional participants were invited to take part in the Delphi survey alone.

There is currently uncertainty regarding the optimal sample size for Delphi method surveys; a wide range of sample sizes have been used within the existing literature, which have often been decided pragmatically (Jorm, 2015; Vogel et al., 2019; Williamson et al., 2017). In this survey, we aimed to recruit sufficient participants to ensure that at least 20 people participated in all three rounds. This balanced our desire to maximize stakeholder representation with the need to ensure the feasibility of timely data analysis and repeat surveys.

PWOE were recruited using a purposive sampling strategy; they were identified either by three of the coauthors or by participants who had already been recruited. Representation was sought from the following services: liaison psychiatry (the hospital-based subspecialty for patients with comorbid mental and physical health needs), drug and alcohol services, intensive or crisis teams, and primary care. A selected sample of leading academic experts in the fields of suicide or substance use research were also invited to participate. In total, 30 PWOE were invited to take part in the survey: 27 were based in the United Kingdom and three were based internationally (the United States: *n =* 2, Australia: *n =* 1). The international participants were invited because they had relevant academic expertise (based on their publication history). PWOE were not compensated for participation.

PWLE were recruited using a convenience and snowball sampling strategy. Members of a pre-existing Patient and Public Involvement (PPI) group with relevant lived experience informed their contacts about the study. Additionally, a post was displayed on UK-based Facebook groups for people with addictions who are in recovery. These posts stated the following inclusion criteria: Participants had to be aged 18 years or older and have lived experience of both substance addictions and suicidal thoughts, suicide attempts, or self-harm. Sixteen PWLE expressed an interest in participating during the specified recruitment period. Before recruitment, potential participants were contacted by phone by a member of the research team to clarify the aims of the study and inclusion criteria. Lived experience participants were reimbursed for participation.

### Data Collection

The telephone interviews were conducted by two of the coauthors and were based on a prespecified topic guide that focused on the following key aspects of the intervention: the inclusion criteria, intervention delivery (including frequency, timing, location, and staff), intervention content, suicide risk management, recruitment, engagement, retention, and the design of a future randomized controlled trial. The interviews were conducted between May and June 2020 and lasted approx. 30–60 min. Three of the coauthors identified the key themes from transcriptions of the interviews.

The Round 1 Delphi survey was developed based on areas of uncertainty that emerged following the telephone interviews and literature review. The survey consisted of 34 items relating to 10-question stems (ESM 2) and five domains: intervention timing, intervention content, intervention delivery, ongoing engagement, and outcomes for a future trial. Further information regarding the rationale for the included items is provided in ESM 1. The Round 1 survey was piloted with an independent researcher and a member of the PPI group who had relevant lived experience. Their feedback was incorporated into its development.

In Rounds 1 and 2, participants were asked to rate each item using a 5-point Likert scale (*strongly agree*, *agree*, *unsure*, *disagree*, *strongly disagree*) to indicate their opinion as to whether the item should be included in the intervention. A section inviting additional comments from participants was included at the end of each survey domain to enable the circulation (in the subsequent round) of a summary of reasoning for items where there was disagreement. In Round 3, to optimize engagement with the survey, the Likert scale was replaced with simplified *Yes/No* options to indicate participants' views as to whether the remaining items should be included in the intervention.

The three rounds of the Delphi survey were conducted online over an 8-week period (July–August 2020). The number of rounds, plan for providing feedback between rounds, and criteria for including and excluding items were decided a priori and were informed by the literature (Hasson et al., 2000; Thangaratinam & Redman, 2005).

The Round 1 survey was sent to all 46 participants (PWOE: *n =* 30, PWLE: *n =* 16) described above, while subsequent surveys were only sent to participants who had completed the previous round. Therefore, the respondents in Round 2 were a subset of those in Round 1 while the respondents in Round 3 were a subset of those in Round 2. In Rounds 2 and 3, when asking participants to rerate items for which consensus had not been reached, participants were provided with the average percentage agreement from the previous round by the panel, stratified by group (PWLE/PWOE). Participants were also provided with a concise summary of the relevant free-text answers from the previous round, and they were able to view their original responses to each survey item before rerating.

The Delphi survey was managed and administered online using Research Electronic Data Capture (REDCap) 9.5.23 tools (Harris et al., 2019), hosted by the University of Bristol. Anonymous identification numbers were assigned to each participant by the REDCap system.

### Data Analysis

Quantitative survey data were analyzed by calculating the pooled percentage of agreement for each item, weighted equally across both groups (PWLE and PWOE). The cutoff used to define agreement was the presence of any agreement; therefore, the responses *agree* and *strongly agree* were combined. Similarly, the cutoff used to define disagreement was the presence of any disagreement; therefore, the responses *disagree* and *strongly disagree* were combined. An agreement threshold of 80% was adopted a priori; items with a pooled agreement rate of 80% or more were included, items with a pooled disagreement rate of 80% or more excluded, and the remaining items were carried over into the next survey for rerating.

Some items provided distinct options relating to the same question; therefore, when one was accepted, the others became redundant and were discarded. An example of this was the options for the timing of the first contact. After the inclusion of the item indicating that this should take place at the time of a first presentation, the items indicating that it should take place 24 hr or 1 week after a presentation were discarded.

Free-text comments provided by participants were separated into key themes. Three of the coauthors discussed the themes at the end of each survey round to decide whether any new survey items needed to be added to subsequent rounds. As a result of this process, an additional item about the need for greater flexibility in the timing of the intervention was added in Round 2.

Where consensus was not obtained on items after Round 3, a decision was made on their inclusion/exclusion in a discussion between the study advisory group (consisting of six of the authors). The rationale behind the decisions made is outlined in the Results section. This approach was selected because the advisory group were able to consider the opinions of both PWLE and PWOE from the Delphi, previously published literature, and potential constraints arising in the planning of the subsequent feasibility study.

## Results

### Participants

Table 1 summarizes the number of people who completed each survey round. The Round 1 survey was completed by 21 PWOE and 15 PWLE. Of those who completed Round 1, 15 (71%) PWOE and 14 (93%) PWLE completed all three surveys. In both groups, respondents were predominantly female [PWOE: *n =* 13 (62%); PWLE: *n =* 9 (60%)]. Of the 21 PWOE who completed Round 1, their main area of expertise was mental health/suicide prevention [*n =* 11 (52%)], addictions [*n =* 9 (43%)], or both [*n =* 1 (5%)]. Their professional backgrounds included clinical psychology/psychiatry [*n =* 10 (48%)], academia [*n =* 9 (43%)], nursing [*n =* 5 (24%)], managerial [*n =* 2 (10%)], and general practice [*n =* 1 (5%)]. All nine academics also had clinical experience: Five had research experience predominantly in the field of suicide/mental health research while four had research experience predominantly in the field of addictions research.

**Table 1:** Number of participants in each panel by Delphi round

Panel	Round 1 (*N*)	Round 2 (*N*)	Round 3 (*N*)	Completion of all 3 rounds (%)
Occupational experience participants	21	17	15	71%
Lived experience participants	15	14	14	93%

### Summary of Rounds

**Figure 1:**
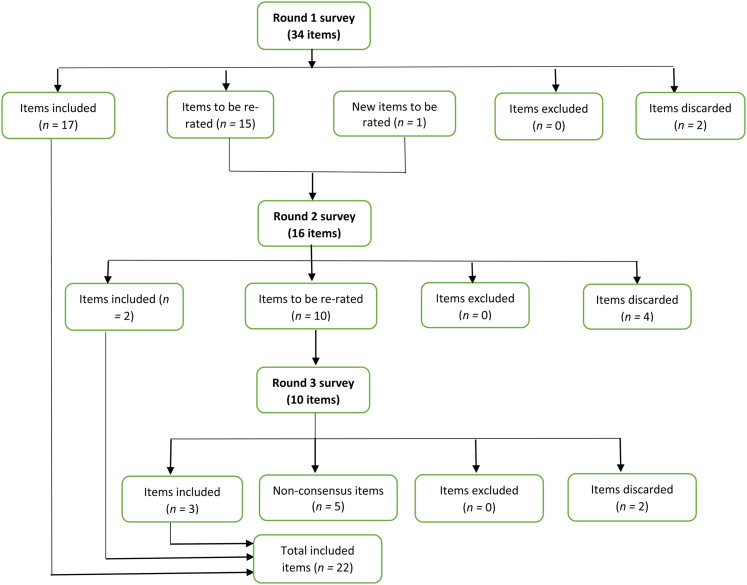
Flowchart of survey item outcomes by Delphi round.

The Round 1 survey included 34 items. After the three rounds, there was a consensus on 22 items (ESM 3). The number of items included, excluded, added, and discarded after each round are outlined in Figure 1. No items reached the threshold level of disagreement to be excluded. A consensus was not obtained for five items relating to two domains (*intervention content* and *ongoing engagement*). As described earlier in the Methods section, a decision about the inclusion/exclusion of these five items was made by the study advisory group.

### Consensus Items

The consensus items specified an intervention that involves face-to-face contact in the emergency department immediately after a psychosocial assessment, followed by an initial phone call 24–72 hr later. Subsequent telephone calls take place weekly for up to one month with personalized reminder texts sent in advance of telephone contact. All contact is provided by Liaison Psychiatry practitioners. During this contact, practitioners will address both self-harm and substance use problems through a range of endorsed items that involve (1) understanding the situation, (2) building motivation for behavioral change, (3) identifying and coping with triggers and urges, and (4) preparing for change (Table 2).

**Table 2:** Included items relating to the intervention content

Intervention component	Item
Understanding the situation	Exploring the patient’s views about underlying reasons for substance use and self-harm
	Exploring the patient’s understanding of the relationship between substance use and mental health
	Providing advice on the relationship between substance use and mental health
Building motivation for behavioral change	Asking the patient to discuss the pros and cons of reducing their use of substances and self-harm behaviors
	Eliciting the patient’s thoughts and feelings about the function of substance use and self-harm in their life
Identifying and coping with triggers and urges	Asking the patient to identify triggers for substance use and self-harm
	Encouraging the patient to record and discuss examples of antecedent/triggers, behavior, consequence in relation to substance use
	Encouraging the patient to record and discuss examples of antecedent/triggers, behavior, consequence in relation to self-harm
	Exploring alternative coping strategies and distraction techniques for managing urges to use substance and self-harm
Preparing for change	Jointly developing a safety plan
	Jointly developing a plan for change with an explicit focus on both substance use and self-harm
	Monitoring the patient's progress in engaging with other community resources, e.g., Alcoholics Anonymous or Samaritans

A consensus was reached on all proposed outcomes for a future randomized controlled trial. One item that specified a patient’s nominated outcome was viewed positively for being patient-centered. However, some highlighted that goal-setting takes time, goals can change, and a patient-centered measure may be challenging to analyze.

### Nonconsensus Items

Two items relating to intervention content did not achieve consensus. These encouraged patients to record either the quantity of substances used or the number of episodes of self-harm in a diary to increase awareness. In all three rounds, there were higher percentages of agreement for these items among PWOE compared with PWLE. Concerns that were raised by some PWLE about the use of diaries included their perceived lack of reliability, the possibility that the recordings might exacerbate feelings of guilt and shame and thereby increase the frequency of self-harm or substance use, and the perception of this being a purely data collection exercise. Many PWOE, however, believed that self-monitoring is an important facet of behavior change and that diaries can serve a useful purpose in informing decisions about future care. In the absence of clear consensus and the inclusion of many other items relating to content, the study advisory group agreed that these items would not be included in the intervention.

The three items relating to the use of gift vouchers to reward patients for taking part in treatment (i.e., a form of contingency management) did not achieve consensus over the three rounds. Many participants thought that, if adopted, this might be the only motivation for people to engage in the intervention. However, they differed in opinion about whether patients might gain from the intervention if not fully committed and intrinsically motivated to change. Some PWLE stated that the voucher would make them feel valued while some participants highlighted challenges with the practicalities of providing vouchers following remote contact. The item with the highest support stated that gift vouchers for the same amount should be provided at the end of each treatment session. These findings were reviewed by the study advisory group. Due to cost constraints, it was decided that during feasibility testing, patients would instead receive a gift voucher upon completion of their final follow-up telephone session.

## Discussion

People with substance use problems who self-harm commonly present to emergency departments, but they lack effective treatment options (Padmanathan et al., 2020). In this Delphi method study, we combined current research evidence with the expertise of people with occupational experience (PWOE) and people with lived experience (PWLE) to inform the core components of a new brief psychosocial intervention for people presenting to the emergency department with self-harm and substance use problems. The developed intervention (ESM 4) consists of weekly follow-up phone calls for up to a month, delivered by Liaison Psychiatry practitioners, in which both self-harm and substance use are explored, and patients are supported in accessing community services. The feasibility of the intervention is initially being tested in an open case series.

### Findings in the Context of the Wider Literature

There is currently a lack of evidence regarding brief psychosocial interventions for people with substance use problems during periods of elevated suicide risk (Padmanathan et al., 2020). Yet, within the field of suicide prevention, there has been growing recognition of the value of suicide or self-harm–specific interventions (Turecki et al., 2019). There has also been increasing interest in brief interventions due to their accessibility (Turecki et al., 2019).

The intervention developed in this study differs from existing brief acute care suicide prevention interventions (Doupnik et al., 2020) due to the inclusion of psychological techniques and psychoeducational content that also address substance use problems and the use of contingency management (in the form of a gift voucher) to improve engagement. The intervention will, therefore, help to develop an understanding of a patient’s situation, build motivation for behavioral change, and prepare for change, in relation to both substance use problems and self-harm. Consequently, the intervention may reduce suicide risk both directly and indirectly through reductions in substance use and engagement with drug and alcohol services.

### Strengths and Limitations

This study included both PWLE and PWOE with a broad range of expertise and experience. The Delphi survey results from each group were weighted equally to account for different sample sizes. The anonymous nature of the survey enabled participants to respond freely while free-text comments at the end of each section provided further insights regarding participants’ ratings and highlighted areas of concern in relation to each domain, which could be fed back to participants. Of participants who completed Round 1, over 70% of PWOE and over 90% of PWLE completed all three rounds. The reasons for the difference in retention rates are unclear but may reflect the reimbursement provided only to PWLE for their time and the demands of PWOE’s work, which were heightened during the COVID-19 pandemic.

While consensus is lacking regarding Delphi method sample/panel sizes, several studies have demonstrated the stability of results with panel sizes of over 20 participants (Jorm, 2015). In our study, the stability of findings between each round may have been affected by the use of smaller panel sizes. However, the results were pooled between panels, and more than half of the survey items were decided upon within the first round itself.

Some aspects of the intervention were determined prior to the survey based on the research literature, stakeholder telephone interviews, and clinical judgment. Consequently, the items included in the survey did not offer choices regarding every aspect of the intervention and may not have reflected participants’ ideal choices. For example, participants may have preferred an intervention of longer duration and greater intensity. Nonetheless, a consensus was rapidly achieved in Round 1 on half of the total number of survey items, and no items were excluded from the survey, indicating that there was minimal disagreement concerning the proposals.

While most survey participants were based within the United Kingdom, thereby potentially limiting the generalizability of the findings to other countries, the intervention was designed primarily to be delivered within the UK National Health Service.

### Conclusion

This Delphi method study incorporated existing research and the expertise of PWLE and PWOE in the development of a new, brief intervention for people presenting to the emergency department with self-harm and substance use problems. An open case series testing the feasibility of the intervention is currently underway.

## Electronic Supplementary Material

The electronic supplementary material is available with the online version of the article at https://doi.org/10.1027/0227-5910/a000933

**ESM 1.** Rationale for decisions made prior to the survey.
**ESM 2.** Delphi survey questions.
**ESM 3.** Summary of survey items for which consensus was obtained.
**ESM 4.** Intervention summary.

## Notes

**Publication Ethics:** This study received ethical approval from the NHS South West – Frenchay Research Ethics Committee (Reference 19/SW/0220).

**Authorship:** Prianka Padmanathan, Conceptualization, Formal analysis, Funding acquisition, Methodology, Writing–original draft. Rachel Cohen, Data curation, Formal analysis, Methodology, Project administration, Writing–original draft. David Gunnell, Funding acquisition, Writing–review & editing. Lucy Biddle, Funding acquisition, Writing–review & editing. Emma Griffith, Funding acquisition, Writing–review & editing. Katie Breheny, Funding acquisition, Writing–review & editing. Matt Hickman, Funding acquisition, Writing–review & editing. Nik Munien, Funding acquisition, Writing–review & editing. Anish Patel, Writing–review & editing. Elaine Crocker, Writing–review & editing. Paul Moran, Conceptualization, Funding acquisition, Methodology, Supervision, Writing–review & editing.

**Open Data:** The data that support the findings of this study are available on request from the corresponding author, Prianka Padmanathan.
