# Supplementary material for: Development of a Brief Intervention for Emergency Department Attendees Presenting With Self-Harm and Co-Occurring Substance Use Problems: A Delphi Method Study
Source: Crisis. 2023 Nov 17;45(4):254–62. doi: 10.1027/0227-5910/a000933 (PMC11370783; doi:10.1027/0227-5910/a000933)
Supplement: Supplementary file 1 [file cri_45_4_254_esm1.pdf]

https://doi.org/10.1027/0227-5910/a000933
© 2023 The Author(s). Distributed as a Hogrefe OpenMind article under the license CC BY 4.0 (https://creativecommons.org/licenses/by/4.0)

ESM 1: Rationale for decisions made prior to the survey

Pre-determined aspects of the intervention

The first decision to be made prior to the survey was about the population to be included. Based on feedback from the telephone discussions, the proposed intervention would be for adults presenting to the hospital with self-harm and concurrent substance use problems; there would not be restrictions on characteristics such as the quantity and frequency of substance use and co-morbid psychiatric diagnoses. We accepted that whilst this would result in heterogeneity within the sample, this approach is in keeping with standard practice in pragmatic RCTs and furthermore would enhance the generalisability of the emergent intervention (Hotopf et al., 1999).

Decisions were also made about the duration and frequency of the intervention and the medium of contact. The intervention would augment standard care and consist of weekly telephone contact during the first four weeks following hospital presentation. The rationale for these decisions was based on: 1) evidence of an exceptionally high risk of suicide during the first month after hospital presentation (Geulayov et al., 2019); 2) Stanley et al.'s (2018) observational study on a safety planning intervention that included weekly follow-up. In this study, a 45% reduction in suicidal behaviours was observed amongst veterans presenting to hospitals in the USA with a suicide-related concern over six months of follow-up (Stanley et al., 2018); 3) O'Connor et al.'s pilot of a similar intervention in the UK, which was considered to be both acceptable and feasible (O’Connor et al., 2022).

https://doi.org/10.1027/0227-5910/a000933
© 2023 The Author(s). Distributed as a Hogrefe OpenMind article under the license CC BY 4.0 (https://creativecommons.org/licenses/by/4.0)

The use of phone calls as the medium of contact in this study was based on: 1) concerns about retention of patients with substance use problems if required to travel regularly to appointments during periods of crisis; 2) shifts to remote working during the COVID-19 pandemic; 3) promising results from several studies of contact-based interventions via telephone for people identified to be at risk of suicide during hospital presentations (Miller et al., 2017; O'Connor et al., 2019; Stanley et al., 2018; Vaiva et al., 2006). Web-based technology was also considered but ruled out by the research team, due to concerns that many individuals in the population of interest would lack reliable access to smartphones, a tablet, or a computer. Letters and postcards were also ruled out early on in the research process, due to limited evidence of their effectiveness in reducing self-harm in Western contexts (Witt et al., 2021).

Rationale for survey items (areas of uncertainty)

Intervention timing

In studies in the general population, suicide prevention interventions that commence during a patient's presentation to clinical services appear to be more effective than those solely delivered post-discharge (Doupnik et al., 2020; Lizardi & Stanley, 2010; Milner et al., 2015). Such an approach may not, however, be acceptable or feasible where people are under the influence of substances when presenting to clinical services. The survey, therefore, asked about preferred timings of the first, second, and subsequent contact.

Previous contact-based interventions have incorporated additional forms of contact, such as postcards and letters (Milner et al., 2015; Witt et al., 2021). Personalised reminder text

https://doi.org/10.1027/0227-5910/a000933
© 2023 The Author(s). Distributed as a Hogrefe OpenMind article under the license CC BY 4.0 (https://creativecommons.org/licenses/by/4.0)

messages in between phone calls were considered to be most feasible, but due to concerns about the risks of data security, these were included as survey items for consideration.

Intervention content

The proposed options for the content of the intervention included techniques commonly used in clinical services for people with substance use problems and/or self-harm. Some of these techniques are recognised features of existing brief interventions and psychological treatments, such as safety planning (Stanley & Brown, 2012), motivational interviewing (Rollnick & Miller, 1995), dialectical behavioural therapy (Linehan, 1993), cognitive behavioural therapy (Hofmann, 2011), and Self-Management and Recovery Training (SMART) Recovery (Beck et al., 2017). Within the Delphi survey, the use of these techniques was specified in relation to both substance use and self-harm or mental health.

Intervention delivery

A choice of delivery of the intervention by Liaison Psychiatry practitioners or a researcher was offered. Although the involvement of peer support workers and the voluntary sector was considered, these latter survey options were deemed unfeasible from a funding and also logistical perspective.

Ongoing engagement

Maintaining engagement is frequently challenging among both people at risk of suicide and people with substance use problems (Lizardi & Stanley, 2010). Furthermore, previous RCTs of interventions for people with comorbid suicide risk and substance use problems have described low engagement (Crawford et al., 2010; Morley et al., 2014). Motivational interviewing is one approach to increasing engagement in psychiatric care

https://doi.org/10.1027/0227-5910/a000933
© 2023 The Author(s). Distributed as a Hogrefe OpenMind article under the license CC BY 4.0 (https://creativecommons.org/licenses/by/4.0)

among people at risk of suicide (Lizardi & Stanley, 2010). Additionally, there is a strong evidence base indicating that rewarding people with substance use problems for meeting therapeutic goals (termed "contingency management") is effective in increasing engagement in treatment, based on the principles of operant conditioning (Lussier et al., 2006; Zajac et al., 2019). This approach has, however, not previously been used within suicide prevention interventions. Gift voucher rewards for participating in the intervention were, therefore, proposed in this study.

Outcomes for a future trial

Finally, the survey included a range of options for potential outcomes for a future RCT. This was included because qualitative research has challenged the meaningfulness of current outcomes measured in trials of interventions for people who self-harm, and highlighted the importance of ascertaining which outcomes matter to those at whom an intervention is aimed (Owens et al., 2020).

References

Beck, A. K., Forbes, E., Baker, A. L., Kelly, P. J., Deane, F. P., Shakeshaft, A., . . . Kelly, J. F. (2017). Systematic review of SMART Recovery: Outcomes, process variables, and implications for research. *Psychol Addict Behav*, *31*(1), 1-20. https://doi.org/10.1037/adb0000237

Crawford, M. J., Csipke, E., Brown, A., Reid, S., Nilsen, K., Redhead, J., & Touquet, R. (2010). The effect of referral for brief intervention for alcohol misuse on repetition of deliberate self-harm: an exploratory randomized controlled trial. *Psychological Medicine*, *40*(11), 1821-1828. https://doi.org/10.1017/S0033291709991899

Doupnik, S. K., Rudd, B., Schmutte, T., Worsley, D., Bowden, C. F., McCarthy, E., . . . Marcus, S. C. (2020). Association of Suicide Prevention Interventions With Subsequent Suicide Attempts, Linkage to Follow-up Care, and Depression Symptoms for Acute Care Settings: A Systematic Review and Meta-analysis. *JAMA psychiatry*, *77*(10), 1021-1030. https://doi.org/10.1001/jamapsychiatry.2020.1586

Geulayov, G., Casey, D., Bale, L., Brand, F., Clements, C., Farooq, B., . . . Hawton, K. (2019). Suicide following presentation to hospital for non-fatal self-harm in the

https://doi.org/10.1027/0227-5910/a000933
© 2023 The Author(s). Distributed as a Hogrefe OpenMind article under the license CC BY 4.0 (https://creativecommons.org/licenses/by/4.0)

Multicentre Study of Self-harm: a long-term follow-up study. *The Lancet Psychiatry*, *6*(12), 1021-1030. https://doi.org/10.1016/s2215-0366(19)30402-x

Hofmann, S. G. (2011). *An introduction to modern CBT: Psychological solutions to mental health problems*. Wiley-Blackwell.

Hotopf, M., Churchill, R., & Lewis, G. (1999). Pragmatic randomised controlled trials in psychiatry. *Br J Psychiatry*, *175*, 217-223. https://doi.org/10.1192/bjp.175.3.217

Linehan, M. M. (1993). *Cognitive-Behavioral Treatment of Borderline Personality Disorder*. Guilford Press.

Lizardi, D., & Stanley, B. (2010). Treatment Engagement: A Neglected Aspect in the Psychiatric Care of Suicidal Patients. *Psychiatric Services*, *61*(12), 1183-1191. https://doi.org/10.1176/ps.2010.61.12.1183

Lussier, J. P., Heil, S. H., Mongeon, J. A., Badger, G. J., & Higgins, S. T. (2006). A meta-analysis of voucher-based reinforcement therapy for substance use disorders. *Addiction*, *101*(2), 192-203. https://doi.org/10.1111/j.1360-0443.2006.01311.x

Miller, I. W., Camargo, C. A., Jr., Arias, S. A., Sullivan, A. F., Allen, M. H., Goldstein, A. B., . . . Boudreaux, E. D. (2017). Suicide Prevention in an Emergency Department Population: The ED-SAFE Study. *JAMA psychiatry*, *74*(6), 563-570. https://doi.org/10.1001/jamapsychiatry.2017.0678

Milner, A. J., Carter, G., Pirkis, J., Robinson, J., & Spittal, M. J. (2015). Letters, green cards, telephone calls and postcards: systematic and meta-analytic review of brief contact interventions for reducing self-harm, suicide attempts and suicide. *Br J Psychiatry*, *206*(3), 184-190. https://doi.org/10.1192/bjp.bp.114.147819

Morley, K. C., Sitharthan, G., Haber, P. S., Tucker, P., & Sitharthan, T. (2014). The efficacy of an opportunistic cognitive behavioral intervention package (OCB) on substance use and comorbid suicide risk: A multisite randomized controlled trial. *Journal of Consulting and Clinical Psychology*, *82*(1), 130-140. https://doi.org/10.1037/a0035310

O'Connor, R. C., Lundy, J.-M., Stewart, C., Smillie, S., McClelland, H., Syrett, S., . . . Simpson, S. A. (2019). SAFETEL randomised controlled feasibility trial of a safety planning intervention with follow-up telephone contact to reduce suicidal behaviour: study protocol. *BMJ Open*, *9*(2), e025591. https://doi.org/10.1136/bmjopen-2018-025591

O'Connor, R. C., Smillie, S., McClelland, H., Lundy, J.-M., Stewart, C., Syrett, S., . . . Simpson, S. A. (2022). SAFETEL: a pilot randomised controlled trial to assess the feasibility and acceptability of a safety planning and telephone follow-up intervention to reduce suicidal behaviour. *Pilot and Feasibility Studies*, *8*(1), 156. https://doi.org/10.1186/s40814-022-01081-5

Owens, C., Fox, F., Redwood, S., Davies, R., Foote, L., Salisbury, N., . . . Thomas, K. (2020). Measuring outcomes in trials of interventions for people who self-harm: qualitative study of service users' views. *BJPsych open*, *6*(2), e22-e22. https://doi.org/10.1192/bjo.2019.93

Rollnick, S., & Miller, W. R. (1995). What is Motivational Interviewing? *Behavioural and Cognitive Psychotherapy*, *23*(4), 325-334. https://doi.org/10.1017/S135246580001643X

Stanley, B., & Brown, G. K. (2012). Safety Planning Intervention: A Brief Intervention to Mitigate Suicide Risk. *Cognitive and Behavioral Practice*, *19*(2), 256-264. https://doi.org/10.1016/j.cbpra.2011.01.001

Stanley, B., Brown, G. K., Brenner, L. A., Galfalvy, H. C., Currier, G. W., Knox, K. L., . . . Green, K. L. (2018). Comparison of the Safety Planning Intervention With Follow-up vs

https://doi.org/10.1027/0227-5910/a000933
© 2023 The Author(s). Distributed as a Hogrefe OpenMind article under the license CC BY 4.0 (https://creativecommons.org/licenses/by/4.0)

Usual Care of Suicidal Patients Treated in the Emergency Department. *JAMA psychiatry*, *75*(9), 894-900. https://doi.org/10.1001/jamapsychiatry.2018.1776

Vaiva, G., Vaiva, G., Ducrocq, F., Meyer, P., Mathieu, D., Philippe, A., . . . Goudemand, M. (2006). Effect of telephone contact on further suicide attempts in patients discharged from an emergency department: randomised controlled study. *BMJ*, *332*(7552), 1241-1245. https://doi.org/10.1136/bmj.332.7552.1241

Witt, K. G., Hetrick, S. E., Rajaram, G., Hazell, P., Taylor Salisbury, T. L., Townsend, E., & Hawton, K. (2021). Psychosocial interventions for self-harm in adults. *Cochrane Database of Systematic Reviews*(4), CD013668. https://doi.org/10.1002/14651858.CD013668.pub2

Zajac, K., Alessi, S. M., & Petry, N. M. (2019). Contingency management approaches. In H. Pickard & S. H. Ahmed (Eds.), *The Routledge Handbook of Philosophy and Science of Addiction* (pp. 455-463). Routledge.
