# Supplementary material for: Development of a Brief Intervention for Emergency Department Attendees Presenting With Self-Harm and Co-Occurring Substance Use Problems: A Delphi Method Study
Source: Crisis. 2023 Nov 17;45(4):254–62. doi: 10.1027/0227-5910/a000933 (PMC11370783; doi:10.1027/0227-5910/a000933)
Supplement: Supplementary file 2 [file cri_45_4_254_esm2.pdf]

https://doi.org/10.1027/0227-5910/a000933
© 2023 The Author(s). Distributed as a Hogrefe OpenMind article under the license CC BY 4.0 (https://creativecommons.org/licenses/by/4.0)

# ESM 2: Delphi survey questions

**Please provide explanations of your responses and/or alternative suggestions to each domain in the free text. This will allow circulation of a summary of reasoning for items where there is disagreement.**

Please note: To ensure this survey is as broadly representative as possible, the term self-harm refers to both suicidal and non-suicidal self-injury.

*Options for answers: strongly agree, agree, unsure/depends, disagree, strongly disagree*

<u>Intervention timing</u>

The **first contact** should take place:

1) Immediately after assessment by the Liaison Psychiatry team, face-to-face
2) 24 hours after presentation to the Emergency Department, via phone
3) 1 week after presentation to the Emergency Department, via phone

**Please provide explanations of your responses and/or alternative suggestions**

The **second contact** will be by phone. This should take place:

1) 24 hours after first contact
2) 72 hours after first contact
3) One week after first contact

**Please provide explanations of your responses and/or alternative suggestions**

**Subsequent** contact should take place:

1) Weekly phone calls for one month
2) Weekly phone calls for one month plus weekly personalised texts in between phone calls

**Please provide explanations of your responses and/or alternative suggestions**

https://doi.org/10.1027/0227-5910/a000933
© 2023 The Author(s). Distributed as a Hogrefe OpenMind article under the license CC BY 4.0 (https://creativecommons.org/licenses/by/4.0)

Content of the intervention

The intervention should involve:

*Understanding the situation*

1) Exploring the patient's views about underlying reasons for substance use & self-harm
2) Exploring the patient's understanding of the relationship between substance use & mental health
3) Providing advice on the relationship between substance use & mental health
4) Encouraging the patient to record the quantity of substances they have used in a diary to increase awareness
5) Encouraging the patient to record the number of episodes self-harm in a diary to increase awareness

*Building motivation*

6) Asking the patient to describe pros and cons of reducing their use of substances and self-harm behaviours
7) Eliciting the patient's thoughts and feelings about the function of substance use & self-harm in their life

*Identifying and coping with triggers and urges*

8) Asking the patient to identify triggers for substance use & self-harm
9) Encouraging the patient to record and discuss examples of antecedent/triggers, behaviour, consequence (ABC) in relation to substance use
10) Encouraging the patient to record and discuss examples of antecedent/triggers, behaviour, consequence (ABC) in relation to self-harm
11) Exploring alternative coping strategies and distraction techniques for managing urges to use substance & self-harm

*Preparing for change*

12) Jointly developing a safety plan
13) Monitoring the patient's progress in engaging with other community resources e.g., Alcoholics Anonymous or Samaritans
14) Jointly developing a plan for change with an explicit focus on both substance use & self-harm

**Please provide explanations of your responses and/or alternative suggestions**

Intervention delivery

The intervention should be delivered by the following staff:

1) First session and follow-up phone calls by researcher
2) First session and follow-up phone calls by Liaison Psychiatry nurses
3) First session by Liaison Psychiatry nurses, follow-up phone calls by researcher

**Please provide explanations of your responses and/or alternative suggestions**

https://doi.org/10.1027/0227-5910/a000933
© 2023 The Author(s). Distributed as a Hogrefe OpenMind article under the license CC BY 4.0 (https://creativecommons.org/licenses/by/4.0)

Ongoing engagement

There is evidence to suggest that rewarding participants with substance use disorders for taking part in treatment can improve their engagement, mental health and likelihood of abstinence.

Which of these approaches should we use:

1) Gift vouchers for same amount at the end of each session
2) Gift vouchers of increasing amounts at the end of each session
3) No gift vouchers or other reward

**Please provide explanations of your responses and/or alternative suggestions**

Outcomes for a trial

These outcomes should be measured when testing the effectiveness of the intervention:

1) Hospital readmissions with self-harm
2) Self-reported suicidal thoughts, self-harm, and suicide attempts
3) Self-reported use of substances
4) Self-reported mental distress
5) The patient's views regarding whether the intervention was as helpful in their recovery
6) Patient nominated outcome with regards to goals for drinking +/- self-harm

**Please provide explanations of your responses and/or alternative suggestions**
