# Supplementary material for: Development of a Brief Intervention for Emergency Department Attendees Presenting With Self-Harm and Co-Occurring Substance Use Problems: A Delphi Method Study
Source: Crisis. 2023 Nov 17;45(4):254–62. doi: 10.1027/0227-5910/a000933 (PMC11370783; doi:10.1027/0227-5910/a000933)
Supplement: Supplementary file 3 [file cri_45_4_254_esm3.pdf]

https://doi.org/10.1027/0227-5910/a000933
© 2023 The Author(s). Distributed as a Hogrefe OpenMind article under the license CC BY 4.0 (https://creativecommons.org/licenses/by/4.0)

ESM 3: Summary of survey items for which consensus was obtained

Section	Question stem	Item	Survey Round
**Intervention timing**	The **first contact** for the intervention should take place:	Immediately after assessment by the Liaison Psychiatry team, face-to-face	1
	The **second contact** will be by phone. This should take place:	24-72 hours after first contact negotiated between the patient and person delivering the intervention	2
	Subsequent contact should take place:	Weekly phone calls for one month plus weekly personalised reminder texts in between phone calls	2
**Content of the intervention**	The intervention should involve (Understanding the situation):	Exploring the patient's views about underlying reasons for substance use & self-harm	1
		Exploring the patient's understanding of the relationship between substance use & mental health	1
		Providing advice on the relationship between substance use & mental health	1
	The intervention should involve (Building motivation for behavioural change):	Asking the patient to discuss the pros and cons of reducing their use of substances and self-harm behaviours	1
		Eliciting the patient's thoughts and feelings about the function of substance use & self-harm in their life	1
	The intervention should involve (Identifying and coping with triggers and urges):	Asking the patient to identify triggers for substance use & self-harm	1
		Encouraging the patient to record and discuss examples of antecedent/triggers, behaviour, consequence in relation to substance use	1
		Encouraging the patient to record and discuss examples of antecedent/triggers, behaviour, consequence in relation to self-harm	1
		Exploring alternative coping strategies and distraction techniques for managing urges to use substance & self-harm	1
	The intervention should involve (Preparing for change):	Jointly developing a safety plan	1
		Monitoring the patient's progress in engaging with other community resources e.g., Alcoholics Anonymous or Samaritans	1
		Jointly developing a plan for change with an explicit focus on both substance use & self-harm	1
**Intervention delivery**	The intervention should be delivered by the following staff:	First session and follow-up phone calls by Liaison Psychiatry nurses	3

https://doi.org/10.1027/0227-5910/a000933
© 2023 The Author(s). Distributed as a Hogrefe OpenMind article under the license CC BY 4.0 (https://creativecommons.org/licenses/by/4.0)

Section	Question stem	Item	Survey Round
**Outcomes for a future trial**	These outcomes should be measured when testing the effectiveness of the intervention:	Hospital readmissions with self-harm	1
		Self-reported suicidal thoughts, self-harm, and suicide attempts	1
		Self-reported use of substances	3
		Self-reported mental distress	1
		The patient's views regarding whether the intervention was helpful in their recovery	1
		The patient's nominated outcome with regards to goals for substance use +/- self-harm	3
