# Supplementary material for: Development of a Brief Intervention for Emergency Department Attendees Presenting With Self-Harm and Co-Occurring Substance Use Problems: A Delphi Method Study
Source: Crisis. 2023 Nov 17;45(4):254–62. doi: 10.1027/0227-5910/a000933 (PMC11370783; doi:10.1027/0227-5910/a000933)
Supplement: Supplementary file 4 [file cri_45_4_254_esm4.pdf]

https://doi.org/10.1027/0227-5910/a000933
© 2023 The Author(s). Distributed as a Hogrefe OpenMind article under the license CC BY 4.0 (https://creativecommons.org/licenses/by/4.0)

ESM 4: Intervention summary

Inclusion criteria: adults who present to hospital with self-harm and concurrent substance use problems

First contact: face-to-face in the emergency department immediately after a psychosocial assessment

Second contact: telephone call 24-72 hours later

Subsequent contact: weekly telephone calls for up to one month after hospital presentation

Other features: delivery by a Liaison Psychiatry practitioner, personalised reminder texts between sessions, provision of a gift voucher after completion of the intervention

Intervention content:

Understanding the situation

1) Exploring the patient's views about underlying reasons for substance use & self-harm
2) Exploring the patient's understanding of the relationship between substance use & mental health
3) Providing advice on the relationship between substance use & mental health

Building motivation

4) Asking the patient to describe pros and cons of reducing their use of substances & self-harm
5) Eliciting the patient's thoughts and feelings about the function of substance use & self-harm in their life

Identifying and coping with triggers and urges

6) Asking the patient to identify triggers for substance use & self-harm
7) Encouraging the patient to record and discuss examples of antecedent/trigger, behaviour, consequence (ABC) in relation to substance use & self-harm
8) Exploring alternative coping strategies and distraction techniques for managing urges to use substances & self-harm

Preparing for change

9) Jointly developing a safety plan
10) Jointly developing a plan for change with an explicit focus on both substance use & self-harm
11) Monitoring the patient's progress in engaging with other community resources e.g., Alcoholics Anonymous or Samaritans
